# Internetbasierte Selbsthilfe bei Einsamkeit aus Sicht der Nutzenden: Eine reflexive thematische Analyse

**DOI:** 10.1007/s00278-026-00881-x

**Published:** 2026-10-06

**Authors:** Noëmi Seewer, Tobias Krieger

**Affiliations:** Abteilung für Klinische Psychologie und Psychotherapie, Institut für Psychologie, Universität Bern, Fabrikstrasse 8, 3012 Bern, Schweiz

**Keywords:** Psychologische Intervention, Kognitive Verhaltenstherapie, Wahrgenommene soziale Isolation, Begleitung, Qualitative Analyse, Psychological intervention, Cognitive behavioral therapy, Perceived social isolation, Guidance, Qualitative analysis

## Abstract

Einsamkeit ist mit vielfältigen gesundheitlichen Belastungen verbunden. Internetbasierte Selbsthilfeinterventionen bieten einen niederschwelligen Ansatz zur ihrer Reduktion, jedoch ist bislang wenig über die Erfahrungen der Nutzenden bekannt. Ziel dieser Studie war es, Erfahrungen mit der internetbasierten Selbsthilfeintervention SOLUS‑D zur Verringerung von Einsamkeit qualitativ zu untersuchen. Die Daten entstammen einer randomisierten kontrollierten Studie zu SOLUS‑D. Nach Abschluss der 10-wöchigen Intervention wurden halbstrukturierte Telefoninterviews mit den Teilnehmenden durchgeführt. Für die vorliegende Analyse wurden 17 Interviews ausgewählt und mittels reflexiver thematischer Analyse ausgewertet. Drei Hauptthemen wurden identifiziert: 1. Nutzende berichteten von Veränderungen in Denken, Fühlen und Handeln, darunter ein bewussterer Umgang mit automatischen Gedanken, mehr Klarheit im Umgang mit Einsamkeitsgefühlen, Aktivierung sowie ein gesteigertes Gefühl von Selbstwirksamkeit. 2. Es zeigte sich die Bedeutung der Begleitung: Automatische Nachrichten wurden eher als Erinnerung erlebt, während menschliche Begleitung Motivation, Sicherheit und das Gefühl, gesehen zu werden, förderte. 3. Es wurde deutlich, dass SOLUS‑D nicht für alle Nutzenden gleichermaßen passend war. Besonders Personen mit dem Wunsch nach direktem sozialem Austausch oder konkreten Kontaktmöglichkeiten erlebten Grenzen des Selbsthilfeformats. Die Ergebnisse verdeutlichen, dass internetbasierte Selbsthilfeinterventionen bei Einsamkeit ein relevantes niederschwelliges Angebot darstellen können. Zugleich unterstreichen sie die Bedeutung einer stärkeren Personalisierung von Inhalten und Begleitung sowie einer gezielten Berücksichtigung individueller und sozialer Einflussfaktoren auf Einsamkeit.

**Zusatzmaterial online:**

Zusätzliche Informationen sind in der Online-Version dieses Artikels (https://doi.org/10.1007/s00278-026-00881-x) enthalten.

## Abstract (en)

Loneliness is associated with a range of adverse health outcomes. Internet-based self-help interventions offer a low-threshold approach to reducing loneliness; however, little is known about users’ experiences. The aim of this study was to qualitatively examine users’ experiences with the internet-based self-help intervention SOLUS‑D for the reduction of loneliness. The data were derived from a randomized controlled trial evaluating SOLUS‑D. Following the 10-week intervention period, semistructured telephone interviews were conducted with the participants. For the present analysis, 17 interviews were selected and analyzed using reflexive thematic analysis. Three main themes were identified. 1: participants reported changes in thinking, feeling and behavior, including increased awareness of automatic thoughts, greater clarity in dealing with feelings of loneliness, behavioral activation and enhanced self-efficacy. 2: the role of guidance emerged as important: automated messages were primarily perceived as reminders, whereas human guidance fostered motivation, a sense of safety and the feeling of being seen. 3: the findings indicated that SOLUS‑D was not equally suitable for all users. In particular, participants seeking direct social interaction or concrete opportunities to form relationships perceived limitations of the self-help format. The results suggest that internet-based self-help interventions can represent a relevant low-threshold option for addressing loneliness. They also highlight the importance for greater personalization of content and guidance specifically addressing individual and social factors that influence loneliness.

Aufgrund des Zusammenhangs von Einsamkeit mit beeinträchtigter Gesundheit werden Einsamkeitsinterventionen, auch in Form internetbasierter Interventionen (IBI’s), angeboten. Trotz der wachsenden Evidenz zur Wirksamkeit von IBI’s weiß man bislang wenig darüber, welche Personen profitieren, über welche Wirkmechanismen die Effekte zustande kommen, und warum sie bei manchen Personen ausbleiben. Solche Erkenntnisse sind jedoch zentral, um Interventionen gezielt einzusetzen und weiterzuentwickeln. Besondere Bedeutung kommt Erfahrungen von Nutzenden einer IBI zur Verringerung von Einsamkeit zu.

Einsamkeit ist über die Lebensspanne hinweg verbreitet und aufgrund ihrer gesellschaftlichen Kosten (Engel et al. 2025) sowie der vielfältigen Zusammenhänge mit der physischen und psychischen Gesundheit (Park et al. 2020) ein hochrelevantes Public-Health-Thema (Mann et al. 2022). Internetbasierte Interventionen haben sich in verschiedenen Studien als niedrigschwellige und vielversprechende Ansatzpunkte erwiesen (Imwinkelried et al. 2024), doch es fehlen vertiefte qualitative Erkenntnisse zu den Erfahrungen der Nutzenden.

## Hintergrund und Fragestellung

Einsamkeit ist ein subjektives, aversives Erleben, das entsteht, wenn eine Diskrepanz zwischen gewünschten und vorhandenen sozialen Beziehungen besteht, entweder hinsichtlich der Quantität oder der Qualität (Peplau und Perlman 1982). Einsamkeit ist von sozialer Isolation abzugrenzen, welche eine objektive Beschreibung des (fehlenden) sozialen Netzwerks einer Person darstellt. Die beiden Konstrukte korrelieren nur gering miteinander (*r* = 0,13; Hilliard et al. 2026): Eine Person kann sich trotz vieler Beziehungen einsam fühlen, während eine andere sich auch mit einem kleineren sozialen Netzwerk nicht einsam fühlt.

Während vorübergehender Einsamkeit ein adaptiver Wert zugeschrieben wird, da sie auf ein unbefriedigtes soziales Bedürfnis hinweist, scheint der anhaltenden oder auch chronischen Einsamkeit genau dieser abhanden gekommen zu sein (Cacioppo et al. 2006). Einsamkeit ist demnach auch mit verschiedenen psychischen Störungsbildern, etwa Depressionen oder Angststörungen, sowie mit einem beeinträchtigten allgemeinen Wohlbefinden assoziiert (Park et al. 2020).

Das kognitive Modell der Einsamkeit (Cacioppo und Hawkley 2009) versucht aufzuzeigen, wie Einsamkeit bestehen bleibt. Darin wird postuliert, dass Menschen, die sich einsam fühlen, eine Hypervigilanz gegenüber potenziell bedrohlichen sozialen Situationen aufweisen sowie an weiteren Stellen der sozialen Informationsverarbeitung Verzerrungen zeigen, wodurch sie sich selbst, andere sowie soziale Interaktionen negativer bewerten (Spithoven et al. 2017). Dem kognitiven Modell zufolge werden diese Wahrnehmungen zusätzlich durch hinderliches soziales Verhalten wie Rückzug oder Passivität verstärkt, wodurch korrektive Erfahrungen ausbleiben und Einsamkeit bestehen bleibt oder sich verstärkt (Cacioppo und Hawkley 2009).

Aufgrund des Zusammenhangs von Einsamkeit mit beeinträchtigter Gesundheit wurde in den letzten Jahrzehnten eine Vielzahl inhaltlich heterogener Einsamkeitsinterventionen entwickelt und evaluiert. Eine kürzlich veröffentlichte Metaanalyse zeigt, dass psychologische Interventionen (z. B. kognitive Verhaltenstherapie oder Achtsamkeitsprogramme) vergleichsweise grössere Interventionseffekte aufweisen als Interventionen, die soziale und emotionale Kompetenzen (z. B. mittels Rollenspielen oder konversationsbasierter Ansätze) trainieren, soziale Unterstützung fördern (z. B. Mentoratsprogramme oder „Freundschafts-Services“) oder auf die Veränderung des sozialen Netzes (z. B. Aktivitätengruppen, Seniorentreffs oder Chöre) abzielen (Lasgaard et al. 2026). Psychologische Interventionen scheinen einen grösseren Effekt auf Einsamkeit zu haben, während soziale Interventionen besser geeignet zu sein scheinen, soziale Isolation zu verringern (Zagic et al. 2021).

Psychologische Interventionen bei Einsamkeit werden zunehmend auch in Form internetbasierter Interventionen angeboten (Imwinkelried et al. 2025). Internetbasierte Interventionen, einsamkeitsübergreifend betrachtet, umfassen verschiedene psychosoziale Ansätze zur Unterstützung bei der Bewältigung psychischer Beschwerden (Berger et al. 2020). Der Begriff „internetbasierte Interventionen“ wird nicht einheitlich verwendet und beinhaltet unterschiedliche Formate, darunter web- oder appbasierte Interventionen, Videotherapie und Chat-Interventionen (Berger et al. 2020). Der vorliegende Artikel konzentriert sich spezifisch auf internetbasierte Selbsthilfe (IBI), wobei psychologische Interventionsinhalte so aufbereitet und über eine Website bereitgestellt werden, dass sie von den Nutzenden selbstständig genutzt werden können (Berger et al. 2020). Solche Interventionen können entweder unbegleitet genutzt oder durch kurze, meist schriftliche Kontakte mit geschulten Personen ergänzt werden, die insbesondere die Motivation und Adhärenz fördern sollen. Diese Ansätze unterscheiden sich somit insbesondere im Grad der Begleitung, verfolgen jedoch beide das Ziel, Betroffene niedrigschwellig zu unterstützen (Berger et al. 2020).

Die IBI erscheinen gerade im Kontext von Einsamkeit besonders sinnvoll. Einsamkeit ist weit verbreitet, geht mit erheblichem subjektivem Leid einher und ist gleichzeitig keine eigenständige psychische Störung. Zudem ist sie häufig mit Scham und Stigmatisierung verbunden, was die Inanspruchnahme klassischer Hilfsangebote erschwert (Barreto et al. 2022). Vor diesem Hintergrund bieten IBI spezifische Vorteile: Sie ermöglichen eine anonyme, kostengünstige sowie zeitlich und örtlich flexible Nutzung und können dadurch auch Personen erreichen, die bestehende Angebote aufgrund von Hemmungen oder anderen Zugangsbarrieren nicht nutzen (Andersson 2016).

Eine kürzlich erschienene Übersichtsarbeit zu internetbasierten Interventionen bei Einsamkeit (Imwinkelried et al. 2024) kommt zum Schluss, dass diese einen vielversprechenden und wahrscheinlich effektiven Ansatz zur Reduktion von Einsamkeit darstellen. So zeigten Käll et al. (2020, 2021) in zwei randomisierten kontrollierten Studien (RCT) in Schweden, dass ein auf kognitiv-verhaltenstherapeutischen Prinzipien basierendes, angeleitetes (*guided*) internetbasiertes Selbsthilfeprogramm (SOLUS) Einsamkeit im Vergleich zu einer Wartekontrollgruppe effektiv reduzieren konnte. Eine darauf aufbauende Intervention (SOLUS-D) erwies sich auch in einem RCT mit deutschsprachigen Teilnehmenden als wirksam (Seewer et al. 2024a). Zudem zeigte sich, dass eine durch Personen (geschulte Studierende in fortgeschrittenem Masterstudium in Psychologie sowie Psycholog:innen auf Doktoratsstufe) begleitete Version einer Variante mit standardisierten, automatisierten Nachrichten überlegen war. Diese Effekte traten unabhängig davon auf, ob die Teilnehmenden die Kriterien einer psychischen Störung erfüllten oder parallel eine Psychotherapie in Anspruch nahmen.

Trotz der wachsenden Evidenz zur Wirksamkeit ist bislang wenig darüber bekannt, welche Personen besonders von solchen Interventionen profitieren, über welche Wirkmechanismen die Effekte zustande kommen, und warum sie bei manchen Personen ausbleiben. Solche Erkenntnisse sind jedoch zentral, um Interventionen gezielter einzusetzen und weiterzuentwickeln.

Vor diesem Hintergrund zielt die vorliegende Studie darauf ab, die Erfahrungen von Nutzenden einer internetbasierten Intervention zur Verringerung von Einsamkeit (Seewer et al. 2024a) zu untersuchen. Hierzu wurden halbstrukturierte Interviews mit Teilnehmenden nach Abschluss der 10-wöchigen Intervention mittels thematischer Analyse ausgewertet.

## Methodik

### Randomisierte kontrollierte Studie und Stichprobe

Die in dieser Studie verwendeten Daten stammen aus einem RCT (Seewer et al. 2024a), das die Wirksamkeit der internetbasierten Selbsthilfeintervention SOLUS‑D zur Reduktion von Einsamkeit sowie den zusätzlichen Effekt menschlicher gegenüber automatischer Begleitung untersuchte. Die Teilnehmenden beider Interventionsgruppen erhielten während der 10-wöchigen Interventionsphase neben SOLUS‑D entweder ein wöchentliches individualisiertes Feedback durch einen E‑Coach (sog. *Guidance*, GU; *n* = 98) oder automatisierte Nachrichten (AN; *n* = 97) mit dem primären Ziel, die selbstständige Nutzung der Intervention zu fördern. Beide Interventionsgruppen wurden mit einer Wartekontrollgruppe (WL; *n* = 48) verglichen.

**Abb. 1:**
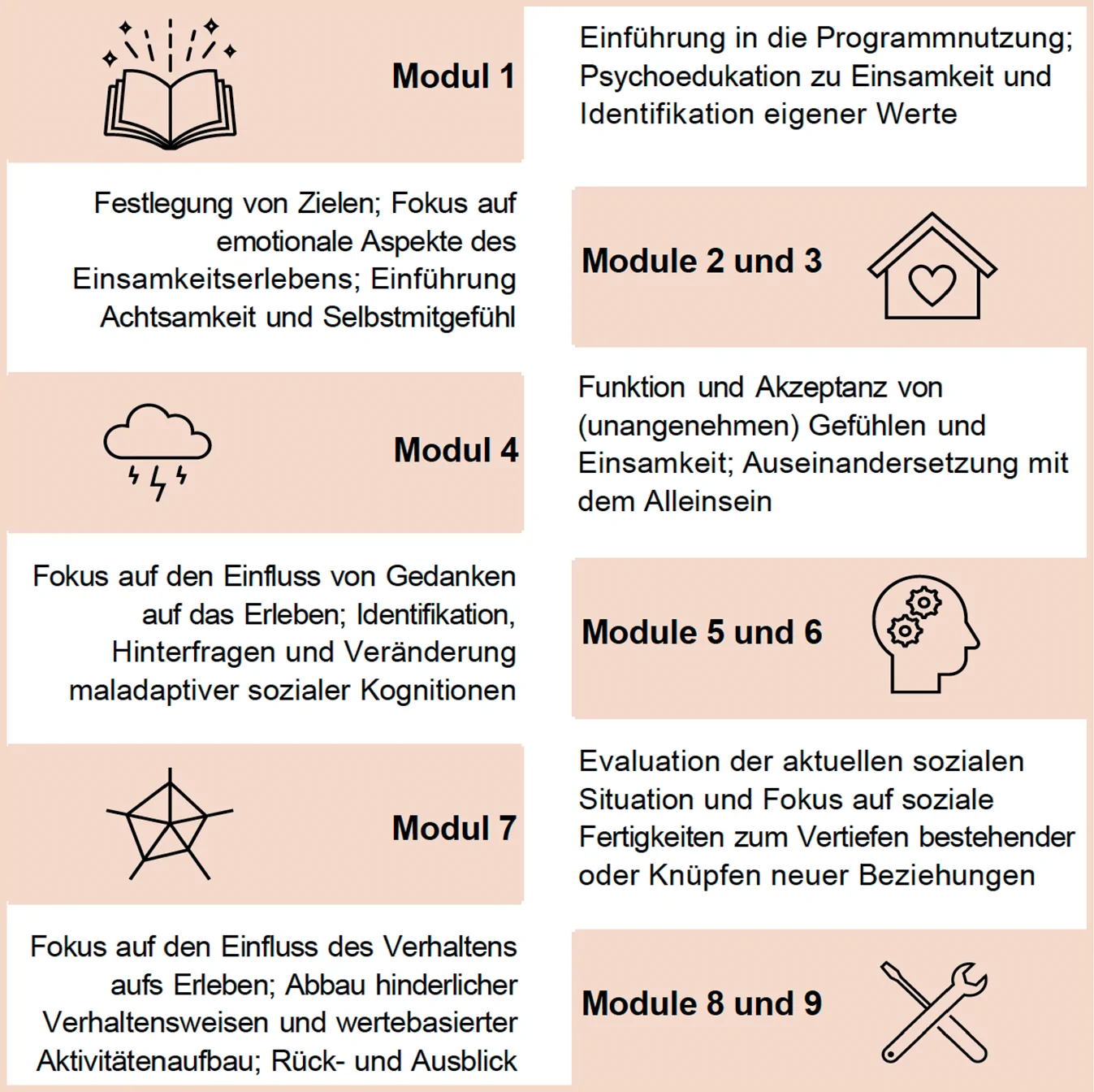
Inhalte der SOLUS-D-Intervention

Bei SOLUS‑D handelt es sich um eine adaptierte Version einer internetbasierten kognitiv-behavioralen Selbsthilfeintervention (Käll et al. 2020); diese besteht aus neun text-, audio- und videobasierten Modulen, die theoretische Inhalte sowie praktische Übungen zur Anwendung im Alltag vermittelten. Eine Übersicht der Inhalte zeigt Abb. 1. Eine detaillierte Interventionsbeschreibung findet sich bei Seewer et al. (2022).

Die Stichprobe der vorliegenden qualitativen Analyse umfasste Teilnehmende beider Interventionsgruppen. Im Rahmen der Postmessung wurde die Bereitschaft zur Teilnahme an qualitativen Interviews erhoben. Die Teilnahme war freiwillig und unvergütet. Insgesamt wurden im Zeitraum von Oktober 2021 bis Juni 2022 42 Interviews durchgeführt, jeweils innerhalb von einer bis 2 Wochen nach Abschluss der Postmessung. Für die vorliegende Studie wurden 17 der 42 Interviews nach dem Prinzip der theoretischen Sättigung ausgewählt. Die Auswahl basierte auf einem Item zur subjektiven Veränderung der Einsamkeit im Vergleich zum Studienbeginn und beinhaltete sowohl Personen mit berichteter Reduktion als auch mit unveränderter oder erhöhter Einsamkeit. Zudem wurde bei der Auswahl darauf geachtet, dass aus beiden Interventionsgruppen jeweils annähernd gleich viele Interviews berücksichtigt wurden.

Das Alter der Teilnehmenden betrug zwischen 26 und 69 Jahre (M = 48,8; SD ± 12,9 Jahre), und 82,3 % der Teilnehmenden waren weiblich. Die Stichprobe setzte sich aus 8 Personen aus der Interventionsbedingung mit menschlicher Begleitung und 9 Personen aus der Bedingung mit automatischen Nachrichten zusammen. Zu Beginn der Interventionsstudie nahmen 17,6 % der Stichprobe eine psychotherapeutische oder psychiatrische Behandlung in Anspruch, und 82,4 % der Personen erfüllten zu diesem Zeitpunkt die Kriterien mindestens einer psychischen Störung (erfasst mit dem Mini-DIPS OA – Diagnostisches Kurzinterview bei psychischen Störungen (Margraf et al. 2017)). Zusätzlich brachten 17,6 % der Teilnehmenden Vorerfahrungen mit anderen internetbasierten Selbsthilfeinterventionen mit. Hinsichtlich dieser Angaben ist die ausgewählte Stichprobe mit der Gesamtstichprobe der interviewten Personen vergleichbar.

### Studiendesign und Datenerhebung

Die Studie wurde als qualitative Untersuchung durchgeführt, weil das Ziel darin bestand, ein vertieftes Verständnis der subjektiven Bedeutung und Erfahrungen der Interventionsnutzenden zu gewinnen, und weniger darauf gerichtet war, vorformulierte Hypothesen zu überprüfen (Creswell und Creswell 2018). Die Datenerhebung erfolgte mittels halbstrukturierter Telefoninterviews, die von zwei geschulten Masterstudierenden und NS durchgeführt wurden. Die Interviews dauerten durchschnittlich 37,2 min (SD ± 12,0 min, Spannweite = 16–65 min). Der Interviewleitfaden (Supplementary Information 1) basiert auf einer für diese Studie angepassten Version des Leitfadens von Urech et al. (2019), der aufbauend auf den Leitfäden von Rozental et al. (2015), Ly et al. (2015) und Lillevoll et al. (2013) entwickelt wurde. Inhaltlich lag der Fokus auf a) der Programmnutzung, b) Erwartungen, Befürchtungen und Hoffnungen vor der Interventionsnutzung, c) Erfahrungen während und nach der Interventionsnutzung, d) positiven und negativen Veränderungen während und nach der Interventionsnutzung, e) technischen Aspekten sowie f) freien Ergänzungen der Teilnehmenden. Mit Einverständnis der Teilnehmenden wurden die Interviews aufgezeichnet und anschliessend wortwörtlich nach dem einfachen Transkriptionssystem (Dresing und Pehl 2015) transkribiert.

### Datenanalyse

Die Transkripte wurden mittels reflexiver thematischer Analyse (RTA) nach Braun und Clarke (2006, 2019) und mithilfe des Programms MAXQDA ausgewertet. Die Analyse erfolgte überwiegend induktiv, ohne vorab festgelegte Kategorien oder ein bestehendes theoretisches Kodierschema. Gleichzeitig war der Analyseprozess durch die Forschungsfrage und den Interviewleitfaden mitstrukturiert, sodass das Vorgehen nicht als vollständig induktiv verstanden werden kann. Der Analyse lag ein essentialistisch-realistischer epistemologischer Ansatz zugrunde, und die Themen wurden auf semantischer Ebene identifiziert, um die explizite Bedeutung der Daten abzubilden und die Erfahrungen bzw. Realität der Teilnehmenden zu berichten. Der Analyseprozess beinhaltete die sechs Phasen nach Braun und Clarke (2006): (1) Vertrautmachen mit den Daten, (2) Generierung initialer Codes, (3) Suche nach Themen, (4) Überprüfung der Themen, (5) Definition und Benennung der Themen sowie (6) Verfassen des Berichts. Die Reflexivität der Forschenden wurde dabei als zentraler Bestandteil des Analyseprozesses verstanden (Supplementary Information 1). Die RTA wurde federführend von NS durchgeführt, die alle sechs Analysephasen eigenständig durchlief. TK machte sich ebenfalls mit dem Datenmaterial vertraut und war insbesondere in die iterativen Phasen 4 und 5 eingebunden. Dabei überprüfte und verfeinerte NS die Themen wiederholt, wobei Rückmeldungen von TK einflossen, bis sie finalisiert wurden. Die Ergebnisse wurden von NS verfasst, und Rückmeldungen von TK wurden dabei berücksichtigt.

## Ergebnisse

**Abb. 2:**
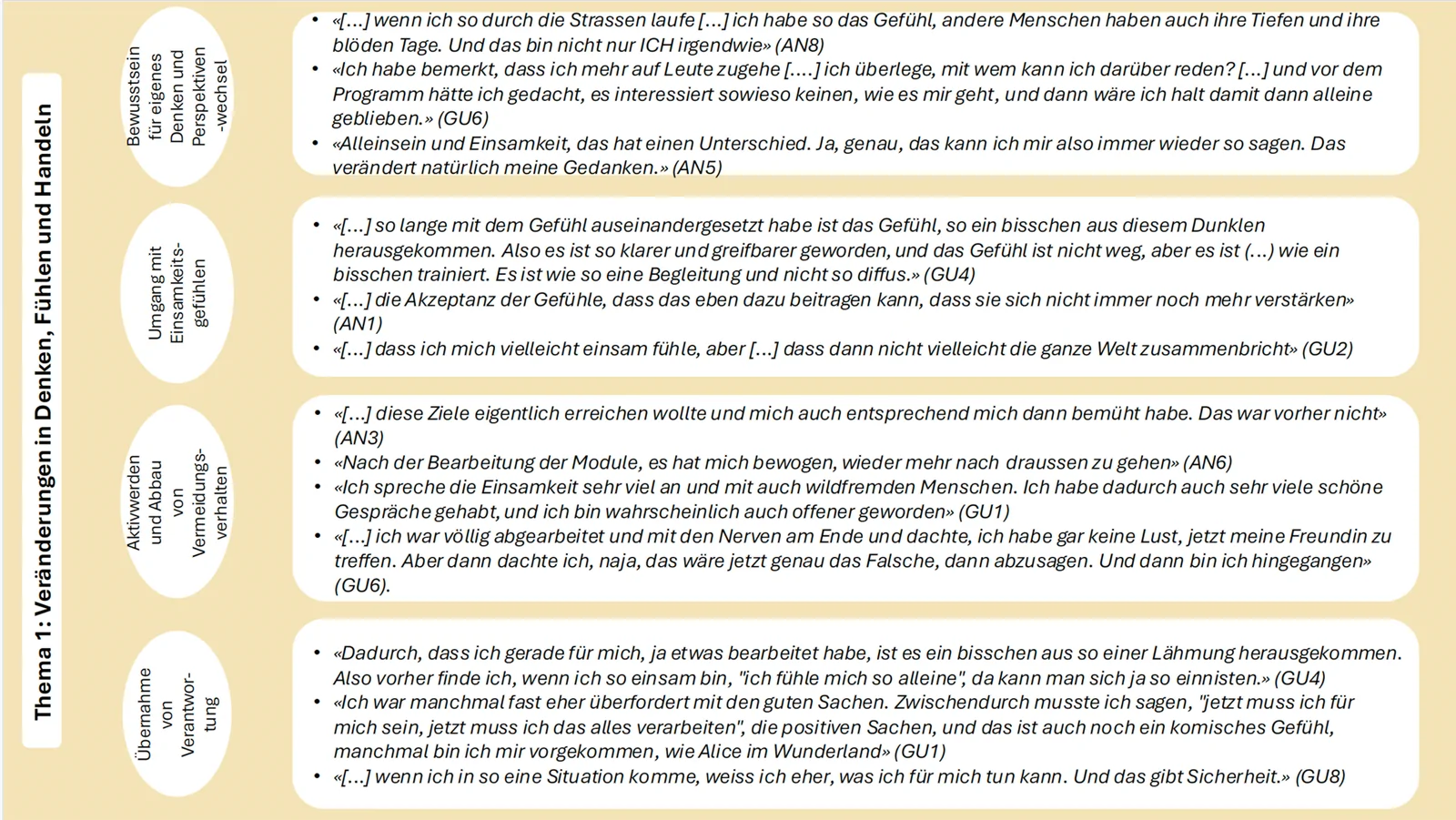
Hauptthema 1 mit Subthemen und dazugehörigen Zitaten von Nutzenden. *GU* Guidance, *AN* automatische Nachrichten

Die thematische Analyse ergab insgesamt drei Hauptthemen mit dazugehörigen Subthemen zu Erfahrungen mit der Selbsthilfeintervention SOLUS‑D. Im Folgenden werden die Hauptthemen und ihre Subthemen (*kursiv*) erläutert. Beispielhafte Zitate aus den Interviews finden sich in den Abb. 2, 3 und 4. Zum besseren Verständnis der Zitate wurden an einigen Stellen kurze Erläuterungen in eckigen Klammern hinzugefügt.

**Abb. 3:**
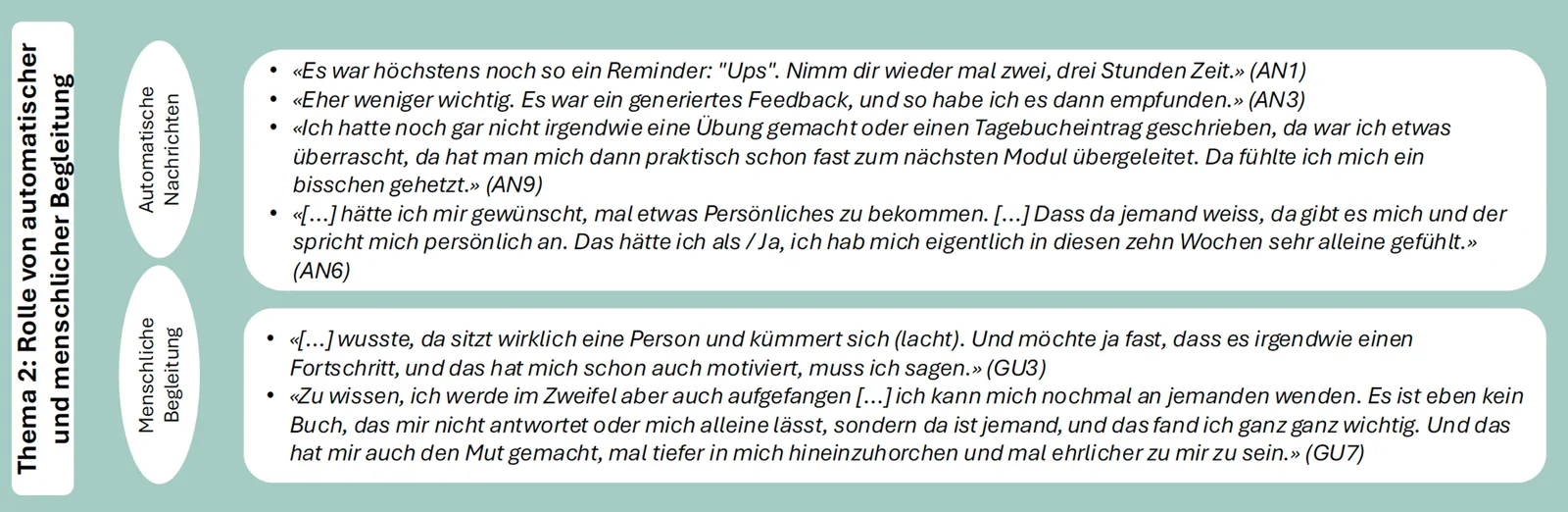
Thema 2 mit Subthemen und dazugehörigen Zitaten von Nutzenden. *GU* Guidance, *AN* automatische Nachrichten

**Abb. 4:**
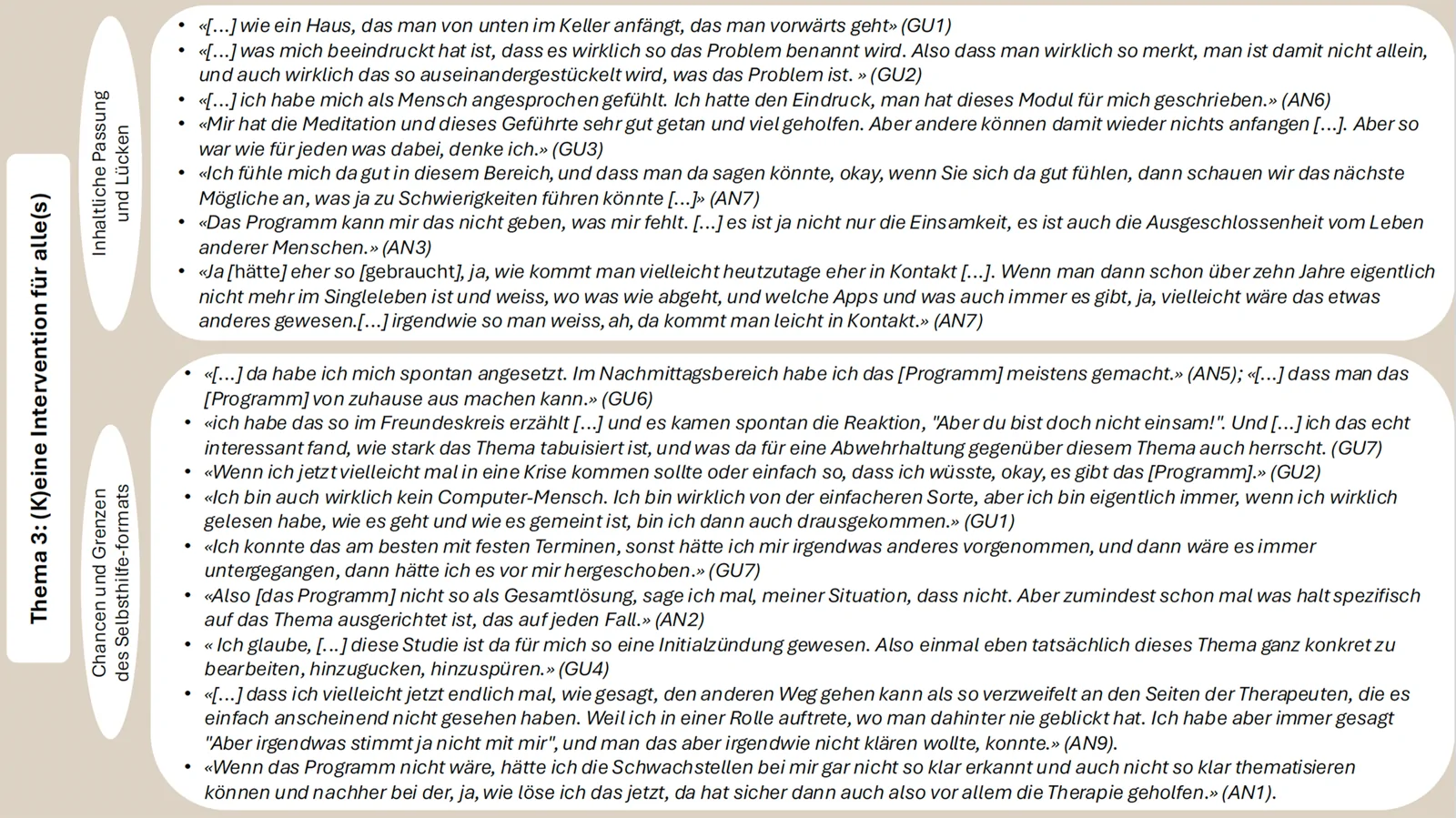
Hauptthema 3 mit Subthemen und dazugehörigen Zitaten von Nutzenden. *GU* Guidance, *AN* automatische Nachrichten

### Hauptthema 1: Veränderungen in Denken, Fühlen und Handeln

Dieses Thema beschreibt vielfältige Veränderungen im Erleben und Verhalten der Nutzenden im Zusammenhang mit der Nutzung der Intervention, die mit einem gesteigerten Gefühl von Selbstwirksamkeit einhergingen. Dabei wurden vier Subthemen identifiziert.

Im Bereich *Bewusstsein für eigenes Denken und Perspektivenwechsel* berichteten Teilnehmende, dass sie sich ihrer automatischen Gedanken und Annahmen über sich selbst, ihre Situation und andere bewusster wurden und diese zunehmend hinterfragen und neu bewerten konnten. Dabei wurde insbesondere neu erworbenes Wissen aus der Intervention als hilfreich für Perspektivenwechsel erlebt.

Auch im *Umgang mit Einsamkeitsgefühlen* zeigten sich Veränderungen. Durch die bewusste Auseinandersetzung mit den Einsamkeitsgefühlen nahm die wahrgenommene Klarheit zu, und das Erleben wurde als weniger diffus beschrieben. Zudem gelang es einigen Teilnehmenden, aufkommende Gefühle besser anzunehmen, wodurch diese weniger belastend erlebt wurden.

Neben kognitiven und emotionalen Veränderungen berichteten die Nutzenden auch von einem verstärkten *Aktivwerden und Abbau von Vermeidungsverhalten*. Das Setzen von Zielen wurde als unterstützend erlebt, und es gelang zunehmend, aus passiven Mustern auszubrechen. Einige Teilnehmende öffneten sich vermehrt gegenüber anderen und arbeiteten aktiv an ihrem Vermeidungsverhalten.

Die *Übernahme von Verantwortung* für die eigene Situation und das eigene Handeln ermöglichte es den Teilnehmenden, korrektive Erfahrungen zu machen und ein gesteigertes Gefühl von Selbstwirksamkeit zu entwickeln.

### Hauptthema 2: Rolle von automatischer und menschlicher Begleitung

Dieses Thema betrifft die Auswirkungen automatischer Nachrichten und menschlicher Begleitung auf die Nutzenden und die Interventionsnutzung. Es wurden zwei Subthemen identifiziert.

Im Subthema *Automatische Nachrichten* wurden die Nachrichten zwar als hilfreiche Erinnerungen wahrgenommen, jedoch häufig als wenig unterstützend erlebt. Ihr Einfluss auf die Motivation zur Programmnutzung wurde insgesamt als eher gering eingeschätzt. Teilweise fühlten sich Teilnehmende dadurch unter Druck gesetzt oder irritiert, insbesondere wenn Nachrichten auf Inhalte Bezug nahmen, die noch nicht bearbeitet worden waren. Zudem wurde das Angebot, sich bei Fragen an das Studienteam zu wenden, kaum genutzt, wodurch sich einige während der Intervention etwas alleingelassen fühlten.

Im Gegensatz dazu wurde die *menschliche Begleitung* als motivierend und unterstützend erlebt. Sie vermittelte Gefühle von Sicherheit, Wertschätzung und Anerkennung. Die E‑Coaches wurden als verständnisvoll und kompetent wahrgenommen, was zu einem erhöhten Verpflichtungsgefühl und einer gesteigerten Motivation zur Nutzung der Intervention beitrug. Das Wissen, dass eine verlässliche Person im Hintergrund verfügbar ist, ermutigte einige Teilnehmende zudem zusätzlich, sich auf neue und herausfordernde Erfahrungen einzulassen.

### Hauptthema 3: (K)eine Intervention für alle(s)

Dieses Thema beschreibt, inwiefern die Intervention SOLUS‑D als geeignet zur Verringerung von Einsamkeit erlebt wurde, und wo ihre Grenzen lagen. Es wurden zwei Subthemen identifiziert.

Im Subthema *Inhaltliche Passung und Lücken* wurde die Möglichkeit geschätzt, sich strukturiert mit der eigenen Einsamkeit auseinanderzusetzen. Mehrere Teilnehmende fühlten sich durch die Inhalte verstanden und gesehen. Während einige es als hilfreich erlebten, passende Inhalte gezielt auszuwählen, hatten andere Schwierigkeiten, allgemeinere Inhalte auf ihre individuelle Situation zu übertragen, und wünschten sich eine stärkere Personalisierung. Insbesondere Teilnehmende mit dem Wunsch nach neuen sozialen Kontakten und direktem Austausch erlebten die Intervention häufig als unzureichend.

Das Subthema *Chancen und Grenzen des Selbsthilfeformats* verdeutlicht, dass die zeitliche und örtliche Flexibilität sowie der flexible Zugriff auf Inhalte als Vorteile wahrgenommen wurden. Gleichzeitig erforderte die Nutzung ein (teilweise zu) hohes Maß an Eigeninitiative und Selbstdisziplin. Während einige Teilnehmende ihre Erwartungen erfüllt sahen oder erste Schritte in Richtung Veränderung machten, stellte die Intervention für andere vor allem eine niederschwellige Möglichkeit dar, sich erstmals gezielt mit ihrer Einsamkeit auseinanderzusetzen. Teilweise wurde sie von Personen, die sich parallel zur Nutzung in einer Face-to-Face-Psychotherapie befanden, auch als sinnvolle Ergänzung beschrieben, da sie sich fokussiert mit dem Thema Einsamkeit auseinandersetzen konnten.

## Diskussion

Ziel dieser Studie war es, die Erfahrungen von Nutzenden mit einer IBI zur Verringerung von Einsamkeit zu untersuchen. Die thematische Analyse identifizierte drei Hauptthemen mit zugehörigen Subthemen und ergänzt damit die bisherige Evidenz zur Wirksamkeit solcher Interventionen. Dass Einsamkeit durch IBI’s reduziert werden kann, wurde bereits in mehreren Studien gezeigt. Die vorliegenden Ergebnisse, insbesondere im Hinblick auf Thema 1, liefern darüber hinaus Hinweise darauf, wie Veränderungen im Einsamkeitserleben zustande kommen können, und stützen Annahmen des kognitiven Modells zur Aufrechterhaltung von Einsamkeit (Cacioppo und Hawkley 2009). Die beobachteten Veränderungen in Denken, Fühlen und Handeln unterstreichen die Bedeutung psychologischer Prozesse, die bei der Reduktion von Einsamkeit berücksichtigt werden müssen. Werden diese nicht adressiert, können sie auch sozialen Interventionen (z. B. der Förderung von Kontaktmöglichkeiten) entgegenstehen (Birken et al. 2025). Für betroffene Personen kann es daher trotz vorhandener Gelegenheiten schwierig sein, soziale Kontakte zu knüpfen, wenn hinderliche Gedanken und Annahmen diesem Verhalten entgegenwirken.

Anhaltende Einsamkeit kann mit einem Gefühl von Kontrollverlust über für die eigene (soziale) Situation relevante Aspekte einhergehen (McKenna-Plumley et al. 2023). Bemerkenswerterweise berichteten einige Nutzende in der aktuellen Studie, dass sie durch die *gezielte* Auseinandersetzung mit dem Thema Einsamkeit mehr Kontrolle über ihre Situation und dadurch auch ein gesteigertes Gefühl von Selbstwirksamkeit entwickelten. Eine systematische Übersichtsarbeit und Metaanalyse weist auf die zentrale Rolle von Selbstwirksamkeit in Studien zu IBI’s bei unterschiedlichen Störungsbildern hin (Behr et al. 2025). Demnach kann Selbstwirksamkeit im Verlauf der Intervention zunehmen und/oder als möglicher Mediator zur Verbesserung der Symptomatik beitragen. Die Interventionsnutzung könnte somit über eine Steigerung der Selbstwirksamkeit zur Symptomreduktion beitragen (Behr et al. 2025). Die Ergebnisse der aktuellen Studie lassen jedoch keine Aussage über die spezifische Rolle von Selbstwirksamkeit im Kontext dieser Einsamkeitsintervention zu, und weitere Forschung hierzu ist nötig, um auch besser zu verstehen, wie und ob sich eine zusätzliche gezieltere Förderung der Selbstwirksamkeit auf das Einsamkeitserleben auswirken würde.

Weiter zeigte sich, dass die menschliche Begleitung eine zentrale Rolle für die Nutzenden spielte. Sie wirkte sich einerseits auf die Motivation zur Interventionsnutzung aus: Die Begleitung wurde als kompetent wahrgenommen, vermittelte Wertschätzung und das Gefühl, gesehen zu werden, und führte zu einem gewissen Verpflichtungsgefühl. Diese Aspekte entsprechen zentralen Komponenten des *Modells der supportive accountability* (Mohr et al. 2011), auf dessen Grundlage die Begleitung in dieser Studie konzipiert war. Andererseits vermittelte die menschliche Begleitung ein Gefühl von Sicherheit, das es den Nutzenden erleichterte, sich auf herausfordernde Inhalte einzulassen und potenziell korrigierende Erfahrungen zu machen. Auch in anderen Studien wurde die Verfügbarkeit einer unterstützenden Person als wichtiger Faktor hervorgehoben (Seittu et al. 2025). Im Gegensatz dazu berichteten Teilnehmende mit automatischer Begleitung häufiger, dass ihnen Aspekte wie Unterstützung, Verständnis und das Gefühl, gesehen zu werden, fehlten. Diese Befunde könnten eine Erklärung für die signifikant geringeren Einsamkeitswerte der Gruppe mit menschlicher Begleitung zum Postzeitpunkt im zugrunde liegenden RCT liefern (Seewer et al. 2024a). Obwohl Interventionen ohne menschliche Begleitung kosteneffizienter sind und potenziell mehr Personen erreichen, könnten sie Nachteile hinsichtlich Nutzungsintensität und damit einhergehender Wirksamkeit aufweisen. Die Entscheidung für oder gegen menschliche Begleitung sollte daher insbesondere im Kontext von Einsamkeit sorgfältig abgewogen und in zukünftigen Kosteneffektivitätsanalysen weiter untersucht werden.

Deutlich wurde, dass der Interventionsinhalt von den Nutzenden in unterschiedlichem Ausmaß als für ihre Bedürfnisse und ihre Situation passend erlebt wurde. Die Passung zwischen Interventionszielen und individuellen Bedürfnissen erwies sich dabei als relevanter Prädiktor für geringere Einsamkeitswerte zum Postzeitpunkt (Seewer et al. 2024b). Insbesondere Teilnehmende, die mehr konkrete Hinweise zum Knüpfen sozialer Kontakte oder direkten Austausch mit anderen erwarteten, erlebten die Inhalte als wenig passend. Dies verdeutlicht die Heterogenität von Einsamkeit als komplexem Phänomen mit unterschiedlichen Ursachen. Entsprechend ist nicht zu erwarten, dass eine einzelne Intervention für alle Betroffenen gleichermaßen geeignet ist. Gleichzeitig weisen die Ergebnisse darauf hin, dass unterschiedliche Formen und Ursachen von Einsamkeit (z. B. emotionale vs. soziale Einsamkeit) stärker berücksichtigt und Interventionen entsprechend differenziert ausgerichtet werden sollten (Walsh et al. 2025).

Insgesamt sprechen diese Befunde dafür, dass eine Personalisierung auf *Inhalt* und *Begleitung* bei Einsamkeit (Hornstein et al. 2023) eine zentrale Bedeutung haben könnte. Auch wenn weniger personalisierte IBI nicht den Bedürfnissen aller Betroffener entsprechen, legen die Erfahrungen der Nutzenden nahe, dass niederschwellige, spezifisch auf Einsamkeit ausgerichtete Angebote, sei es eigenständig oder als komplementäre Intervention, eine wichtige Rolle spielen können.

Zudem zeigte sich, dass wahrgenommene Stigmatisierung im sozialen Umfeld ein mögliches Hindernis für das Hilfesuchverhalten darstellt. Sowohl erlebte als auch antizipierte Stigmatisierung im Zusammenhang mit Einsamkeit kann den Zugang zu Unterstützung erschweren (Langenkamp und Schobin 2024). Die Aussagen einzelner Nutzenden deuten zudem darauf hin, dass eine spezifische Berücksichtigung von Einsamkeit im therapeutischen Kontext als hilfreich erlebt wurde. Da anhaltende Einsamkeit jedoch keine diagnostizierbare, klar umrissene Störung ist, können neben dem Erkennen von Einsamkeit auch die damit verbundene Handlungssicherheit sowie die strukturelle Verankerung und Finanzierung entsprechender Unterstützungsangebote erschwert sein. Vor diesem Hintergrund erscheinen einsamkeitsspezifische, niederschwellige IBI vielversprechend.

Die Ergebnisse dieser Studie sind unter Berücksichtigung mehrerer Limitationen zu interpretieren. Die Teilnahme an den Interviews war freiwillig, weshalb anzunehmen ist, dass sich eher Personen beteiligten, die die Studienteilnahme und die Intervention positiv erlebt haben. Um dieser Verzerrung entgegenzuwirken, wurden bei der Auswahl der Interviews sowohl Teilnehmende mit berichteter Verbesserung als auch mit unverändertem oder verschlechtertem Einsamkeitserleben berücksichtigt. Zudem wurden die Interviewer:innen geschult und darauf sensibilisiert, eine offene Gesprächsatmosphäre zu schaffen und zu betonen, dass es keine richtigen oder falschen Antworten gibt. Dennoch kann sozial erwünschtes Antwortverhalten im Rahmen telefonischer Interviews nicht ausgeschlossen werden. Eine weitere potenzielle Verzerrungsquelle stellt die Rolle der Erstautorin dar, die maßgeblich an der qualitativen Analyse beteiligt war. Zur Minimierung dieses Einflusses erfolgten eine kontinuierliche Reflexion eigener Annahmen und Erfahrungen sowie ein regelmäßiger Austausch mit dem Zweitautor. Im Rahmen der reflexiven thematischen Analyse wird die aktive Rolle der Forschenden explizit anerkannt und Subjektivität nicht als vermeidbar verstanden. Dies impliziert jedoch, dass unterschiedliche Forschende bei der Analyse desselben Datenmaterials zu teilweise unterschiedlichen Ergebnissen gelangen können.

## Fazit für die Praxis

- Einsamkeit ist ein komplexes Phänomen mit individuellen und sozialen Anteilen und sollte entsprechend auf mehreren Ebenen adressiert werden.
- Aufgrund ihrer Niederschwelligkeit, Anonymität und Flexibilität können IBI’s besonders geeignet sein, Menschen mit Einsamkeit zu erreichen – insbesondere vor dem Hintergrund von Stigmatisierung und einem reduzierten Hilfesuchverhalten. Strukturierte IBI’s können Einsamkeit wirksam reduzieren und gehen mit Veränderungen auf kognitiver, emotionaler und behavioraler Ebene sowie einer Stärkung der Selbstwirksamkeit einher.
- Menschliche Begleitung erhöht Motivation und Nutzungsintensität und unterstützt Nutzende dabei, sich auf herausfordernde Veränderungsschritte einzulassen.
- Internetbasierte Interventionen sind nicht für alle Betroffenen gleichermaßen geeignet, stellen jedoch ein relevantes niederschwelliges Angebot dar und sollten, wenn möglich, zukünftig mehr personalisiert (Inhalt, Begleitung) werden.

## Supplementary Information

Interviewleitfaden und Reflexivitätsstatement

## Data Availability

Die im Rahmen dieser Studie verwendeten Daten stehen Dritten zum Schutz der Vertraulichkeit und Privatsphäre der Teilnehmenden nicht zur Verfügung, da sie sensible Informationen enthalten.
