# Supplementary material for: Internet-based self-help for loneliness from the users’ perspective: A reflexive thematic analysis
Source: Psychotherapie (Heidelb). 2026 Oct 6;71(6):452–60. [Article in German] doi: 10.1007/s00278-026-00881-x (PMC13714050; doi:10.1007/s00278-026-00881-x)
Supplement: Supplementary file 1 [file 278_2026_881_MOESM1_ESM.docx]

[Supplementary Information 1	1](#_Toc240985926)

[Interview-Leitfaden	1](#_Toc240985927)

[Supplementary Information 2	5](#_Toc240985928)

[Reflexivitätsstatement	5](#_Toc240985929)

# Supplementary Information 1

## Interview-Leitfaden

Vor dem Beginn des Interviews

1. Begrüssen und bedanken, dass sich die Person Zeit nimmt, um das Interview durchzuführen. Nachfragen, ob der Zeitpunkt immer noch passend ist. Z.B.

*«Guten Tag. Ich bin XY vom SOLUS-Studienteam. Wir sind heute für ein telefonisches Interview verabredet. Und passt Ihnen der Zeitpunkt noch?»*

- Falls «nein, passt nicht», neuen Termin für das Interview vereinbaren.

«*Vielen Dank, dass Sie sich die Zeit nehmen, mit mir dieses Interview durchzuführen. Wie bereits per
E-Mail angekündigt, möchten wir Sie in diesem Interview genauer zu Ihrer Meinung zum und Ihren Erfahrungen mit dem SOLUS-Programm befragen.*

2. Person informieren, dass das Interview aufgezeichnet wird, damit es im Anschluss verschriftlicht werden kann. Informieren, dass die Aufnahme gelöscht wird, sobald das Interview verschriftlicht wurde. Die Person informieren, dass das Aufnahmegerät noch nicht läuft. Z.B.

«*Damit ich mich während des Interviews ganz auf Sie konzentrieren kann, würde ich unser Gespräch gerne aufzeichnen. Nach dem Interview werde ich diese Aufnahme verschriftlichen und alle Angaben, die Rückschluss auf Ihre Person zulassen, werden entfernt. Danach wird die Aufnahme unwiderruflich gelöscht. Ist das in Ordnung für Sie? Im Moment läuft das Aufnahmegerät noch nicht. Sobald wir bereit sind, um mit dem Interview zu beginnen, werde ich das Aufnahmegerät einschalten und noch einmal sagen, dass das Interview aufgezeichnet wird*.»

Einführung ins Interview

«*Das Interview wird ca. 30-50 Minuten dauern, dies hängt ganz davon ab, wie viel Sie mir mitteilen möchten.*»

***NUR BEI SCHWEIZER:INNEN****: «Da es sich um ein halb-standardisiertes Interview handelt, würde ich Ihnen die Fragen gerne auf Hochdeutsch stellen und wäre dankbar, wenn Sie diese auch auf Hochdeutsch beantworten könnten. Wäre das für Sie in Ordnung? Wenn nicht, können wir es gerne auch auf Schweizerdeutsch führen.»*

*«Mir ist sehr wichtig zu betonen, dass es keine richtigen oder falschen Antworten gibt. Mich interessiert, Ihre ganz persönliche Meinung und Ihre Erfahrungen.»*

*«Wenn Sie etwas nicht verstehen, fragen Sie bitte jederzeit nach.»*

*«Haben Sie bis hierhin Fragen? Falls nicht, dann können wir gerne mit dem Interview beginnen.»*

Interview

*Wie bereits eingangs erwähnt, werde ich dieses Gespräch aufzeichnen. Sind Sie damit einverstanden?* (Person **muss ja sagen**, andernfalls **darf nicht aufgezeichnet werden.** Person noch einmal darüber informieren, dass die Aussagen im Interview so verarbeitet werden, dass nicht auf sie zurückgeschlossen werden kann. Zudem aufklären, dass Aufzeichnung lediglich erstellt wird, um Interview in anonymisierter Form zu transkribieren und danach umgehend gelöscht wird. Willigt Person nicht ein, Interview ohne Aufnahme durchführen, kurze Notizen machen.)

*Vielen Dank. Dann würden wir mit dem Interview starten und ich würde gerne mit einer Frage zur aktuellen Nutzung des internetbasierten Selbsthilfeprogramms einsteigen.*

1 Nutzung des Programmes

1. Verwenden Sie das internetbasierte Selbsthilfeprogramm SOLUS weiterhin?
   a. Wenn ja: Wie häufig verwenden Sie das Programm ungefähr?
   b. Wenn nein: Weshalb nicht?

*Vielen Dank. Die nächsten Fragen beziehen sich auf den Zeitraum, bevor Sie mit der Nutzung des SOLUS-Programmes begonnen haben.*

2 Vor der Nutzung des Programmes

1. Was hat Sie dazu bewogen, an unserer Studie teilzunehmen? Welche Aspekte der Studie haben Sie besonders angesprochen?
2. Was waren Ihre Erwartungen/Hoffnungen an das internetbasierte Selbsthilfeprogramm?
3. Hatten Sie Zweifel oder Bedenken in Bezug auf das internetbasierten Selbsthilfeprogramm?
   a. Wenn ja: Welche?
   b. Wenn nein: Warum nicht?

*Vielen Dank für Ihre Ausführungen. Die nächsten Fragen beziehen sich auf die 10 Wochen, während derer Sie das internetbasierte Selbsthilfeprogramm im Rahmen der Studie genutzt haben.*

3 Nach/während der Nutzung des Programmes (10 Wochen)

1. Was ist (bisher) Ihre stärkste Erinnerung an das internetbasierte Selbsthilfeprogramm?
2. Wie viele der 9 Module haben Sie insgesamt bearbeitet? Wenn Sie nicht alle bearbeitet haben, was waren die Gründe dafür?
3. Was hat Ihnen am internetbasierten Selbsthilfeprogramm besonders gefallen? Was war aus Ihrer Sicht besonders hilfreich? Nennen Sie bitte, wenn möglich, auch Beispiele. (*z.B. eher allgemeine Aspekte oder bestimmte Übungen/Inhalte*)
4. Was hat Ihnen am internetbasierten Selbsthilfeprogramm nicht gefallen, war hinderlich, nicht hilfreich, negativ oder enttäuschend? Nennen Sie bitte wenn möglich auch hier Beispiele. (*z.B. eher allgemeine Aspekte oder bestimmte Übungen/Inhalte*)
5. Was war Ihres Erachtens die hilfreichste und die am wenigsten hilfreiche Übung respektive Inhalt im Programm? Weshalb?
6. Haben Sie im internetbasierten Selbsthilfeprogramm etwas vermisst/hat Ihnen etwas gefehlt?
   a. Wenn ja: Was hat Ihnen gefehlt? Oder: Was hätte das Selbsthilfeprogramm für Sie effektiver oder hilfreicher gemacht?
7. Wie haben Sie das wöchentliche Feedback zu den Modulen erlebt? Hat Sie dieses in irgendeiner Weise beeinflusst?
   a. Nur für Guidance: Wie war es für Sie, dass ihr E-Coach Ihre Aktivitäten im Programm mitverfolgen konnte? Hat Sie dies in irgendeiner Weise beeinflusst? Wenn ja, inwiefern?
   b. Nur für Guidance: Wie haben Sie die Begleitung durch den E-Coach erlebt? (evtl. schon in Frage 7 beantwortet)
8. Wie haben Sie das Programm genutzt? Z.B. zu einem fixen Zeitpunkt, mehrmals pro Woche? Können Sie mir das etwas erläutern?

*Als nächstes würde ich Ihnen gerne einige Fragen zu Ihrem Befinden und* ***positiven*** *Veränderungen stellen, die während/nach der Nutzung des internetbasierten Selbsthilfeprogrammes aufgetreten sind.*

4 Positive Effekte/Veränderungen

1. Wie fühlen Sie sich im Moment ganz im Allgemeinen?
2. Wie fühlen Sie sich im Moment in Bezug auf Ihre Einsamkeitsgefühle?
3. Hat sich in Bezug auf Ihre Einsamkeitsgefühle etwas zum Positiven verändert, seit Sie mit der Nutzung des Programms begonnen haben?
   i. *Folgende Nachfragen können hilfreich sein: Handeln, fühlen oder denken Sie anders als zuvor?; Welche Hinweise oder Ideen, vielleicht auch über sich selbst, haben Sie durch die Teilnahme erhalten?; Haben andere Menschen Ihnen gesagt, dass sich etwas an Ihnen verändert hat?*
   b. Wenn ja: Können Sie mir beschreiben, was sich verändert hat? Können Sie hierzu Beispiele nennen?
   c. Wenn ja: Führen Sie diese Veränderung auf Aspekte des Selbsthilfeprogrammes zurück?
      i. Wenn ja: auf welche?
      ii. Wenn nein: Was denken Sie, hat aus Ihrer Sicht zu einer Verbesserung beigetragen?
4. Wie beeinflusste das Selbsthilfeprogramm ihr alltägliches Leben?
5. Können Sie sich an eine oder mehrere Situationen erinnern, in denen Sie sich anders verhalten haben aufgrund der Inputs, die Sie aus dem SOLUS-Programm erhalten haben? (evtl. schon in Frage 3 beantwortet)
   a. Wenn ja: Können Sie diese Situationen genauer beschreiben?

*Vielen Dank. Nun würde ich Ihnen gerne einige Fragen zu* ***negativen*** *Veränderungen stellen, die während/nach der Nutzung des internetbasierten Selbsthilfeprogrammes aufgetreten sind.*

5 Negative Effekte/Nebenwirkungen

1. Sind während der Nutzung des internetbasierten Selbsthilfeprogrammes neue Beschwerden aufgetreten, die Sie vorher nicht hatten oder nicht kannten?
   a. Wenn ja: Welche?
   b. Wenn ja: Führen Sie das Aufkommen der neuen Beschwerden auf die Nutzung des Selbsthilfeprogramms zurück?
2. Haben sich während den 10 Wochen, in welchen Sie das Selbsthilfeprogramm nutzten, Ihre Einsamkeitsgefühle oder andere Beschwerden/Symptome verstärkt?
   a. Wenn ja: Was hat sich verstärkt?
   b. Wenn ja: Führen Sie die Verschlechterung auf das Selbsthilfeprogramm zurück?
3. Gab es während den 10 Wochen, in welchen Sie das Selbsthilfeprogramm nutzten, bestimmte Ereignisse, die zu einer Verschlechterung Ihres Befindens geführt haben?
   a. Wenn ja: Welche?
   b. Wenn ja: Stehen diese Ereignisse im Zusammenhang mit der Nutzung des Selbsthilfeprogramms?

*Gerne würde ich Ihnen nun einige Fragen zur Nutzung des Programmes als solches stellen.*

6 Technik

1. Was könnte Ihrer Meinung nach am SOLUS-Programm in Bezug auf die Technik verbessert werden? Gab es Aspekte, die bei der Verwendung nicht praktisch/nicht intuitiv/schwierig waren?
2. Gab/Gibt es Dinge, die Sie am SOLUS-Programm gestört haben?
   a. Wenn ja: Welche?

*Zum Schluss würde ich Ihnen gerne noch einige etwas allgemeinere Fragen stellen.*

7 Sonstiges

1. Glauben Sie, mit dem internetbasierten Selbsthilfeprogramm die passende Unterstützung erhalten zu haben, um sich mit den eigenen Einsamkeitsgefühlen auseinandersetzen zu können?
   a. Falls nein: Welches wäre für Sie eine passendere Form der Unterstützung gewesen?
   b. Wenn Ja: Möchten/werden Sie das Programm weiterhin zu nutzen?
2. Haben Sie irgendwelche Vorschläge, wie wir das internetbasierte Selbsthilfeprogramm verändern, anreichern oder verbessern könnten?
3. Gibt es etwas, das noch nicht angesprochen wurde, Sie mir aber gerne noch sagen möchten?

Abschluss und Verabschiedung

*Wir sind nun am Ende des Interviews angelangt. Vielen Dank, dass Sie sich die Zeit für dieses Gespräch genommen und meine Fragen beantwortet haben. Wie war das für Sie?*

*Wie Sie der Studieninformation entnehmen konnten, sind wir auch daran interessiert, wie sich die Nutzung des Programmes langfristig auswirkt. Deshalb kontaktieren wir Sie in ca. 4 und 8 Monaten erneut per E-Mail und bitten Sie, weitere Fragebogen auszufüllen. Diese sind deutlich kürzer als die bisherigen. Zudem würden wir Sie in 4 Monaten gerne für eine Kurzversion des diagnostischen Interviews, das wir bereits vor Beginn der Studie mit Ihnen durchführen durften, kontaktieren. Gerne möchte ich Sie auch noch darauf hinweisen, dass Sie das Programm weiterhin nutzen können.*

*Sollten Fragen oder Unklarheiten auftreten, können Sie sich jederzeit gerne per E-Mail bei Noëmi Seewer melden.*

*Passt das so für Sie oder haben Sie noch offene Fragen oder Anmerkungen?*

*Dann bedanke ich mich noch einmal dafür, dass Sie sich Zeit genommen haben für dieses Gespräch und wünsche Ihnen alles Gute.*

# Supplementary Information 2

## Reflexivitätsstatement

NS trägt die Hauptverantwortung für die Durchführung der Analysen. Bereits vor dem RCT, aus dem die hier verwendeten Daten stammen, führten NS und TK eine unveröffentlichte Pilotstudie zur SOLUS-D-Intervention durch. Dabei waren NS und TK auch an der Übersetzung und Aufbereitung der Intervention beteiligt. Basierend auf den Rückmeldungen der Pilotstudie adaptierten TK und NS die Intervention, die dann im RCT verwendet wurde. NS und TK haben gemeinsam den Interviewleitfaden erstellt und NS war für die Schulung der Masterstudierenden, die an der Durchführung und Transkription der Interviews beteiligt waren zuständig. NS und TK haben somit eine grosse Vertrautheit mit der SOLUS-Intervention und der Studie.

NS und TK reflektierten während des Analyseprozesses wiederholt, wie ihre Beteiligung an der Entwicklung und Durchführung der Studie die Wahrnehmung und Interpretation des Datenmaterials beeinflussen könnte. Dabei berücksichtigten sie insbesondere die Möglichkeit, positive Erfahrungen mit der Intervention stärker wahrzunehmen und kritische oder negative Erfahrungen weniger stark zu gewichten. Eigene Annahmen und alternative Interpretationen wurden sowohl untereinander als auch mit weiteren Forschenden der Universität Bern kritisch diskutiert.

Die Interviews wurden telefonisch geführt. Dies kann sich auf die Reichhaltigkeit der Daten ausgewirkt haben, da nonverbale Aspekte (v.a. Gestik, Mimik) nicht mitberücksichtigt werden könnten und somit das Verständnis der Aussagen mitbeeinflusst haben kann.

NS und TK sind zudem psychotherapeutisch tätig. Ihr klinischer Hintergrund könnte die Analyse dahingehend beeinflusst haben, dass psychologisch bzw. therapeutisch relevante Prozesse im Datenmaterial besonders rasch wahrgenommen wurden, während andere Aspekte möglicherweise weniger Beachtung fanden. Dieser mögliche Einfluss wurde im Verlauf der Analyse wiederholt reflektiert.
